# Advancing workplace mental health equity in post-apartheid South Africa: an intersectional mixed-methods study

**DOI:** 10.3389/fpubh.2026.1794110

**Published:** 2026-05-11

**Authors:** Anurag Shekhar, Musawenkosi D. Saurombe

**Affiliations:** College of Business and Economics, Department of Industrial Psychology and People Management, University of Johannesburg, Johannesburg, South Africa

**Keywords:** black tax, gender equity, intersectionality, mixed-methods research, social determinants of health, South Africa, workplace mental health, workplace well-being

## Abstract

**Background:**

Workplace mental health is a global public health priority, yet evidence from African contexts remains limited. This study examined workplace well-being through an intersectional social determinants lens in a South African organisational context to identify equity-relevant disparities and explain the mechanisms producing them.

**Methods:**

An explanatory sequential mixed-methods design was employed. Quantitative survey data (*n* = 87) assessed mental well-being (WEMWBS), perceived stress (PSS-4), work engagement (UWES-3), life satisfaction (SWLS), and flourishing across demographic groups (gender, race, generation, income, education). Qualitative data comprised one focus group discussion (*n* = 9) and semi-structured interviews (*n* = 10) exploring lived experiences of workplace well-being, stress, and engagement. Data were analysed using non-parametric tests and reflexive thematic analysis, with integration guided by an Intersectional Social Determinants Model. Intersectionality is explored primarily through qualitative inquiry and mixed-methods integration rather than quantitative interaction modelling, given sample size constraints.

**Results:**

Gender significantly predicted mental well-being (*p* = 0.015), with women reporting lower scores. Income significantly predicted mental well-being (*p* = 0.033), life satisfaction (*p* = 0.011), and perceived stress (*p* < 0.001), with higher-income employees reporting better outcomes across all three domains. Race and generation showed no significant main effects. Qualitative findings helped explain these patterns through four mechanisms: (1) gendered unpaid labour and caregiving strain reduced women’s mental well-being, particularly among Black and Mixed ancestry (South Africa) women at specific intersections; (2) Black tax (kin-based financial obligations) appeared to constrain income’s protective effects, sustaining financial strain despite salary parity; (3) restrictive masculine norms constrained men’s emotional expression and help-seeking, masking distress in survey measures; and (4) race operated through intersectional mechanisms rather than as uniform main effects.

**Conclusion:**

Workplace well-being in post-apartheid South Africa is shaped by intersecting social determinants rather than isolated demographic factors. Culturally responsive, equity-oriented interventions must address structural caregiving burdens, kin obligations, and masculine norm constraints while redistributing workplace demands and resources. Income’s protective effects are socially mediated: Black tax constrains financial benefit even at higher salary levels, challenging the assumption that economic resources operate uniformly in structurally unequal settings. The Intersectional Social Determinants Model offers an organising framework for future inquiry in similar contexts.

## Introduction

Workplace mental health is a global public health priority, with meaningful work acting as a protective factor while poor working conditions worsen mental health outcomes (1, 2). Meaningful work can act as a protective factor for health, while bad working conditions or unemployment can worsen mental health conditions (1). These micro-level job factors are linked to broader social patterns that determine health outcomes (3). Psychological well-being is a strong predictor of productivity, attendance, and general organisational functioning (4). This makes workplace well-being a population health concern with implications for mental health equity, productivity, and societal resilience.

The State of the Global Workplace report shows persistently low engagement and widespread negative emotions among employees, including daily stress and sadness (5). About 41% of employees report experiencing significant stress, with stress levels almost 60% higher in organisations with poor management practices (5). Low employee engagement is estimated to cost the global economy 8.9 trillion dollars, equal to 9% of global GDP (5). Mental health conditions like depression and anxiety cost the world 1 trillion dollars annually in lost productivity (5). Loneliness affects 20% of workers and is reported at higher levels for remote workers (5). These macro-level economic data highlight the global urgency of workplace well-being interventions (1, 6, 7).

However, the evidence base for workplace well-being interventions remains geographically uneven. Most studies on well-being and positive psychology occur in Western countries, often called WEIRD societies (Western, Educated, Industrialised, Rich, and Democratic) (8). Western countries represent over 78% of studies in the science of well-being (8). Only a small amount of research comes from the African continent (9). This gap means that many models ignore the role of culture and context (9). Many early studies in Africa used tools made in the West without checking if they fit local realities (9). The international landscape of research is dominated by a few nations (10). Interventions designed in high-income settings may not translate effectively into contexts shaped by structural inequality, extended-family obligations, and limited mental health services.

South Africa provides a unique context for studying workplace well-being because it is one of the most unequal countries in the world (11). Race is the biggest contributor to this inequality (12). The legacy of apartheid still shapes economic differences today (12). Historically disadvantaged groups have higher rates of mental ill-health (13). Black professionals also face the pressure of Black tax, an informal system of financial support for extended family members (14, 15). Black tax is a daily reality for almost every Black South African (15). A survey by the South African Depression and Anxiety Group found that 75% of employees think about work when they are not there, and half reported feeling unhappy on Monday mornings (16). These macro-level factors influence how individuals experience social obligations at the meso level.

To address workplace well-being inequities, intersectionality theory offers a powerful framework. Intersectionality examines how overlapping identities such as race and gender interact to shape lived experiences (17). Standard models often erase the specific location of women of colour, as feminism often focuses on white women and antiracism on men of colour (17). This leaves women of colour at a disadvantage in both movements (17). Understanding these dynamics is vital for meso-level analysis of social positions in workplace contexts.

Social determinants of mental health frameworks emphasise that well-being is shaped by structural and environmental factors (18). Integrating intersectionality with social determinants perspectives is particularly valuable in Global South settings where workplace stress is interwoven with broader structural realities. For example, income may not confer equal psychological benefit if financial resources are redistributed through extended-family obligations, and gendered norms may place unequal caregiving burdens on women while discouraging men from expressing vulnerability.

Methodologically, mixed-methods approaches are well suited to this agenda because they can capture both patterned disparities and the lived mechanisms underlying them. Combining quantitative survey data with qualitative interviews allows researchers to address health disparities by including the voices of targeted populations (19). This design enables examination of how meso-level social patterns connect to micro-level job outcomes.

This study makes one central theoretical contribution. It argues that the protective value of economic resources is not fixed but socially mediated: in post-apartheid South Africa, income does not operate as a straightforward individual resource because culturally embedded obligations, most notably Black tax, redistribute financial resources through kin networks and constrain their psychological benefits. This shifts workplace well-being research towards a more contextually grounded understanding of what counts as a resource in structurally unequal settings.

### Study aims

Building on this rationale, the present study examined workplace well-being through an intersectional social determinants lens in a South African organisational context. The study had three aims:

1. To quantify patterns of workplace well-being outcomes (mental health, stress, engagement, life satisfaction, flourishing) across demographic positions in a South African workplace, as a mapping step to identify where disparities are concentrated.
2. To qualitatively elucidate the lived mechanisms through which macro-level social determinants shape meso-level intersectional positioning and translate into micro-level job demands, resources, and well-being outcomes.
3. To generate contextually grounded implications for workplace mental health equity in post-apartheid South Africa.

An explanatory sequential mixed-methods design was employed to first identify demographic patterning quantitatively, then explain and contextualise these patterns through qualitative exploration of employees’ lived experiences.

### Conceptual framework: intersectional social determinants model of workplace well-being

**Figure 1:**
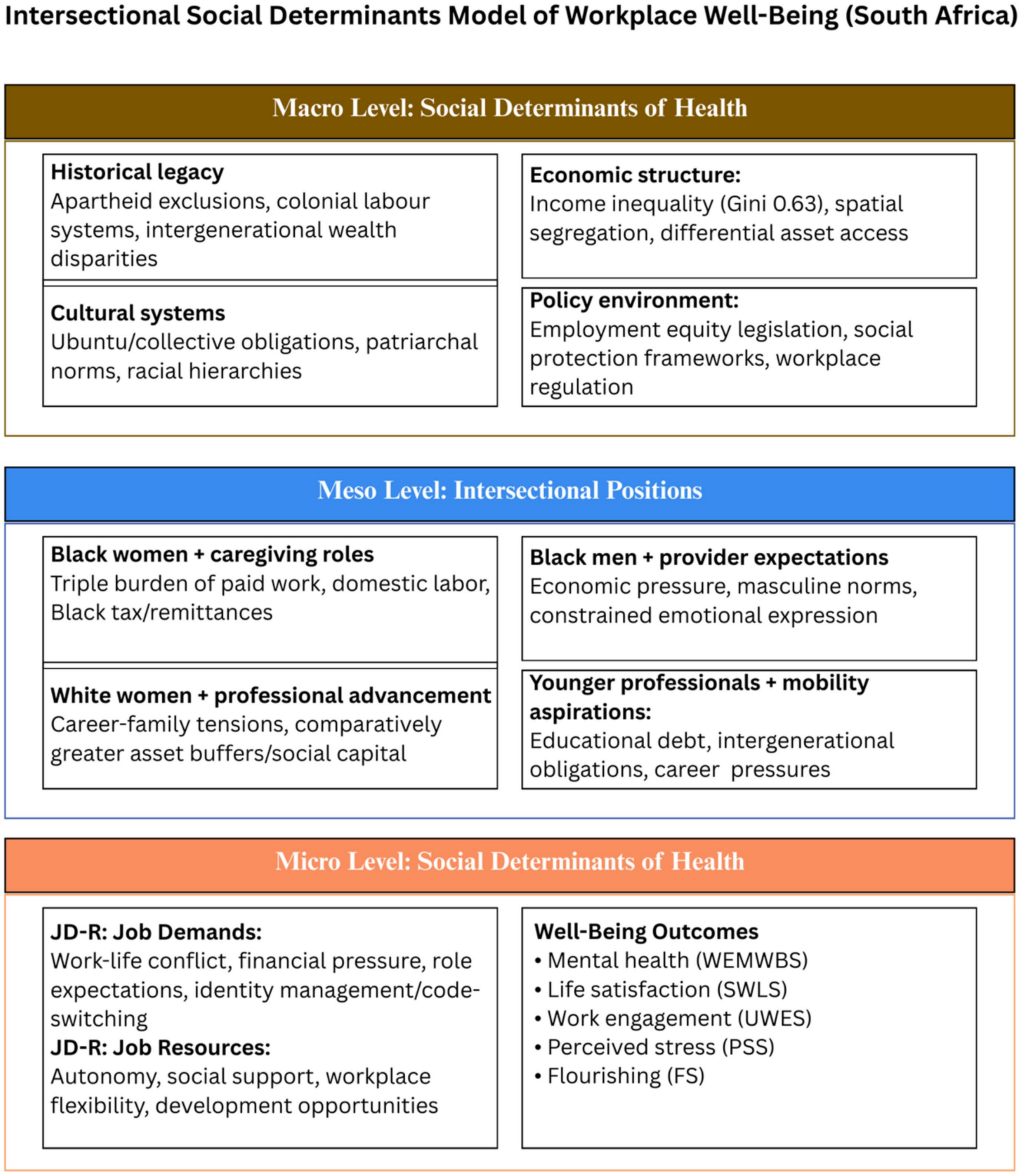
Intersectional social determinants model of workplace well-being (South Africa). This framework integrates social determinants of mental health (18), intersectionality theory (17), and the Job Demands–Resources model (20, 21) to explain how macro-level structural conditions shape meso-level intersectional positioning, which translates into micro-level workplace experiences and well-being outcomes.

This study is organised around an Intersectional Social Determinants Model of Workplace Well-Being (Figure 1). The framework integrates three established theoretical perspectives: intersectionality theory (17), social determinants of mental health (18), and the Job Demands–Resources model (20, 21).

The framework brings together three theoretical perspectives for the study’s inquiry, rather than proposing a new theory in its own right. At the macro level, historical legacy (apartheid exclusion, intergenerational wealth disparities), cultural systems (patriarchal norms, collective obligations like ubuntu), economic structure (income inequality, differential asset access), and policy environment create structural conditions. At the meso level, these determinants shape intersectional positions through demographic categories and role expectations. The framework identifies four illustrative positions: Black women with caregiving roles experiencing triple burdens of paid work, domestic labour, and kin caregiving; Black men navigating provider expectations and constrained emotional expression; White women managing career-family tensions; and younger professionals balancing debt, intergenerational obligations, and career pressures. At the micro level, intersectional positioning translates into daily workplace experiences through job demands (work-life conflict, financial pressure, role expectations) and job resources (autonomy, social support, flexibility, development opportunities). The balance between demands and resources shapes well-being outcomes: mental health, life satisfaction, work engagement, perceived stress, and flourishing (22).

The framework’s value is heuristic: it organises the study’s multi-level inquiry and supports integration of quantitative and qualitative findings. The study’s substantive contribution, developed later in the Discussion, concerns how culturally embedded obligations shape the relationship between income and well-being.

### Literature review

This literature review is organised around the three-level Intersectional Social Determinants Model of Workplace Well-Being (Figure 1) to build a systematic case for the present study. The review first establishes workplace mental health as a public health and organisational imperative, then examines the evidence gap in Global South contexts. It subsequently unpacks each level of the framework: macro-level social determinants shaping structural conditions, meso-level intersectional positioning producing differential exposure to burdens and resources, and micro-level workplace processes through which demands, resources, and interventions influence well-being outcomes.

### Workplace mental health as a public health and organisational imperative

Mental health is an increasingly important topic in workplace contexts (1, 6, 23, 24). Common disorders like depression and anxiety are the leading cause of sickness absence (1, 6, 23). These conditions are often treatable and preventable, yet they create significant economic and personal costs to society (23). About 15% of working-age adults have a mental disorder at any point in time (1). Depression and anxiety alone cause 12 billion work days to be lost every year (1). Management training can support workers and reduce emotional distress by recognising distress in teams and managing job stressors (1). Employee engagement is a major factor in overall life evaluations, with engaged workers experiencing better daily emotions and lower stress (5). Psychological well-being also predicts attendance and productivity (4).

Taken together, these trends support a public health rationale for workplace-based prevention: rather than relying solely on clinical services, workplaces offer scalable settings to reduce distress before problems become entrenched.

### The evidence gap in global South workplace research

Despite this urgency, research on well-being in the Global South remains quite limited (8). South African research on well-being is growing but remains small (9). Indigenous African values often focus on interconnectedness and community, differing from Western individualist models that focus on the self (9). Only 5% of psychological research focuses on the non-Western world (8).

This evidence gap is not simply methodological but conceptual. Much dominant workplace well-being literature assumes individualised stress models and universal intervention mechanisms, yet Global South contexts often involve structurally embedded stressors requiring broader, equity-sensitive framings. This gap justifies examining workplace well-being through a meso-level analysis that attends to local identities and structural positions.

### Macro level: social determinants of mental health in South Africa

#### Historical legacy and economic inequality

The macro level of the framework (Figure 1) positions workplace well-being as an outcome of upstream structural conditions including historical legacy, cultural systems, economic structure, and policy environment. In South Africa, these determinants are inseparable from the country’s history. Apartheid created a segregated society with limited access to jobs and healthcare (13). This system was designed to prevent Black people from building wealth across generations (13). Income inequality actually increased in the years after 1994 (25). Race explains about 41% of this inequality (12). These historical and economic factors are macro-level drivers of how people evaluate their lives and experience workplace well-being.

#### Cultural systems and economic structure

Cultural systems in South Africa also impact well-being outcomes at the macro level. Ubuntu is a philosophy that means “a person is a person through other persons” (26). This values interdependence and community over the individual (27). Many South Africans believe that life has a spiritual dimension, with natural and supernatural worlds viewed as connected (28). Ancestors are often seen as guides for the living (28). However, cultural norms can also create pressures. Women often do more unpaid family work than men (27). These macro cultural norms set the stage for meso-level social obligations.

The economic structure of the country is another major macro-level factor in well-being. High unemployment is a chronic risk to health in South Africa (25). The unemployment rate for youth reached over 55% in 2019 (11). This creates an “employment paradox” where people have high hopes but few jobs (11). For those with jobs, income is a major predictor of satisfaction (29). Well-being rises with income even at high levels (29). Income changes impact mental health and well-being for working adults (30). These macroeconomic factors influence the financial transfers that occur at the meso level.

### Meso level: intersectionality and positioned well-being

#### Intersectionality as framework

The meso level of the framework (Figure 1) emphasises that macro-level determinants are not experienced uniformly but are mediated through intersecting social identities and structural positions. At the meso level, intersectionality explains how identities create unique experiences of well-being. Black women in South Africa face a double disadvantage (27). They often earn less than white women and Black men (27). They are also more likely to lead households in poverty (27).

#### Gendered well-being and caregiving

Gendered well-being is a key finding at this meso level. Research confirms a sizeable gender well-being gap (31). Women across many countries report poorer mental health than men (31). They have more bad mental health days and more restless sleep (31). In South Africa, women have historically reported lower life satisfaction than men (11). Economic progress for women has not yet closed this mental health gap (31). Economic shocks often have a more adverse impact on marginalised groups like women (31). These meso-level gender differences are rooted in macro-level patriarchal structures that assign disproportionate caregiving and domestic labour responsibilities to women.

#### Racialized financial obligations: black tax

Black tax is a significant meso-level pressure for Black professionals in South Africa. It is the money that workers send to support their extended families (14). It helps family members survive poverty engineered by apartheid (15). For the sender, Black tax is often experienced as a financial burden (15). It can reduce a person’s ability to save and invest for themselves (15). Some also see it as an act of ubuntu that keeps families together (15).

Black tax may help explain how income translates into well-being because it reduces the personal resources available to the worker. Conceptually, Black tax can be understood as a socially enforced redistribution mechanism: it operates not through individual choice but through relational obligation embedded in historical exclusion and cultural reciprocity. Unlike discretionary charitable giving, Black tax carries social sanction—failing to contribute risks reputational damage, family conflict, and community ostracism. This distinguishes it from generic financial strain and positions it as a structural constraint on individual resource accumulation. As a theoretical construct, Black tax sits at the intersection of labour economics, cultural obligation, and psychological wellbeing—a mechanism through which macro-level inequality reproduces itself at the level of individual financial security despite professional advancement. This meso-level positioning mediates the relationship between macro-level economic inequality and micro-level financial pressure.

#### Masculine norms and emotional suppression

Masculine norms also shape the experiences of men at the meso level. Traditional Zulu culture requires men to be emotionally strong and worthy of respect (28). A man must not show vulnerability in front of his family (32). He is expected to be the provider and decision maker (32). These norms can mask distress because men are reluctant to ask for help (28). They may see seeking mental health support as a sign of weakness (33). Instead of talking, men may use substances or show aggression as an outlet (32). These meso-level norms prevent men from utilising micro-level job resources effectively, constraining access to emotional support and help-seeking.

### Micro level: job demands–resources and well-being outcomes

#### The job demands–resources model

The micro level draws on the Job Demands–Resources model (20, 21), which explains how job demands—workload, emotional pressure, role conflict—deplete well-being, while resources—autonomy, support, flexibility—buffer against strain. Critically, what counts as a demand or resource is not fixed: it is shaped by the meso-level positions employees occupy.

Understanding how meso-level intersectional positions translate into differential exposure to micro-level demands and access to resources is essential for explaining workplace well-being disparities in post-apartheid South Africa.

#### Synthesis and study rationale

Despite conceptual foundations established in this review, significant gaps remain. First, there is limited quantitative evidence mapping workplace well-being disparities across intersecting demographic categories in South African organisational contexts. Second, mechanisms through which macro-level determinants translate into micro-level well-being outcomes through meso-level intersectional positioning have not been qualitatively examined in African workplace settings. Third, the extent to which income, gender, race, and other demographic factors predict well-being outcomes in post-apartheid South African workplaces (and whether these relationships operate through culturally specific mechanisms such as Black tax, gendered caregiving, or masculine norm enforcement) remains empirically unclear.

The present study addresses these gaps through an explanatory sequential mixed-methods design that first quantifies demographic patterning in well-being outcomes, then qualitatively elucidates the lived mechanisms shaping these patterns within a South African organisational context.

## Methods

### Study design

This study employed an explanatory sequential mixed-methods design (19, 34) to examine workplace well-being through an intersectional social determinants lens. Quantitative survey data were first collected to identify patterns in employee well-being outcomes across demographic groups, testing whether gender, race, generation, income, and education were associated with mental well-being, perceived stress, work engagement, life satisfaction, and flourishing. Qualitative data were then collected through a focus group discussion (FGD) and semi-structured interviews to explain and contextualise the quantitative findings by exploring employees’ lived experiences.

The sequential structure allowed qualitative inquiry to be purposively directed towards explaining quantitative patterns, strengthening integration and supporting development of contextually grounded implications for workplace mental health equity.

### Setting and context

#### Organisational setting

The study was conducted within a single organisation in Johannesburg, South Africa. The organisation operates in the healthcare services sector and employed 108 people at the time of data collection, with a workforce that was demographically diverse across race, gender, age cohort, income, and education level. This diversity reflected the post-apartheid composition of urban professional workplaces and made the setting appropriate for intersectional inquiry. The organisation was selected through purposive criterion sampling: it met three conditions necessary for the study’s aims—demographic diversity enabling subgroup comparison, the lead researcher’s insider access enabling trust-based qualitative engagement, and organisational permission for both survey and interview data collection.

A single-organisation design was deliberate: it provided the depth and contextual specificity necessary to explore how macro-level social determinants translate into lived workplace experiences, which a multi-site survey design would not have captured. The trade-off is limited generalisability, which the study acknowledges explicitly by positioning findings as exploratory and hypothesis-generating rather than population-level estimates.

### Participants and recruitment

#### Quantitative phase

All employees of the organisation were invited to complete the survey. Participation was voluntary and anonymous. 87 participants out of 108 employees completed the survey. The survey was administered in English and included demographic variables (gender, race, age cohort, income category, education level) to support intersectionality-informed subgroup analyses. Race categories were based on participant self-identification using common South African classifications; we use the term mixed ancestry to refer to participants who may be classified as ‘Coloured’ in South African demographic reporting.

**Figure 2:**
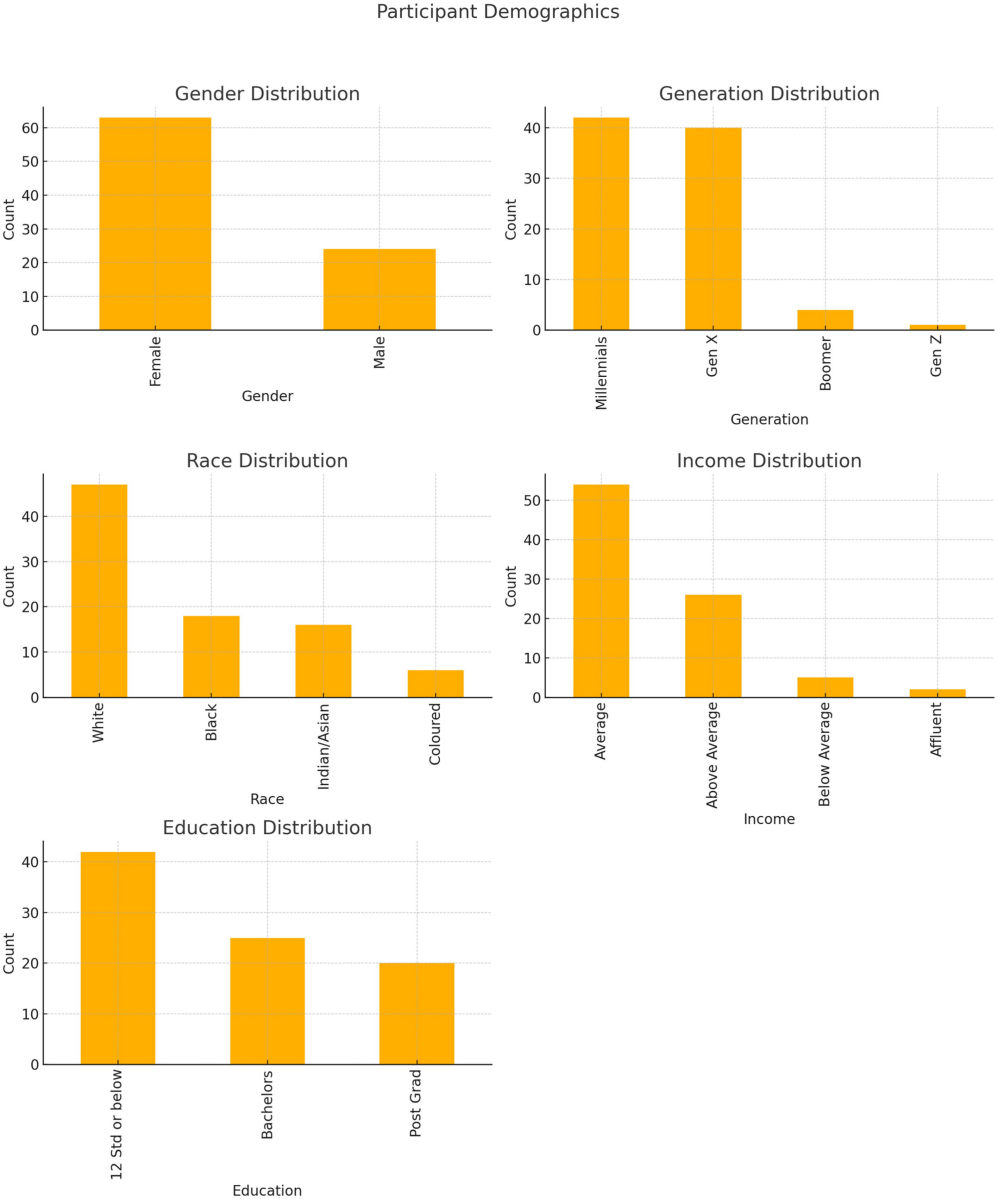
Participant demographics showing distributions across gender, generation, race, income, and education (quantitative sample, *n* = 87).

Figure 2 summarises the demographic distribution. The sample included representation across gender, race (Black African, White, Indian/Asian, Mixed ancestry), generation (Millennials, Generation X, Generation Z, Baby Boomers), income categories, and education levels (Matric, Diploma, Bachelor’s, Master’s). This diversity was essential for examining well-being disparities through an intersectional lens, although sample size limited statistical power for modelling complex interaction effects.

#### Qualitative phase

Survey respondents were invited to participate in the qualitative phase. Participants were purposively selected using maximum variation sampling to ensure representation across key demographic categories and organisational roles. A total of 19 employees participated in the qualitative part of the study: focus group discussion (*n* = 9) and individual semi-structured interviews (*n* = 10). Recruitment ceased when the research team judged that sufficient information power had been achieved: meaning that the sample provided adequate depth and specificity to address the study’s aims within the defined scope (35). This assessment was based on: (1) strong analytic structure (clear theoretical framework guiding inquiry), (2) high participant specificity (purposive sampling ensuring representation across key intersections), and (3) dense, recurring patterns in later interviews indicating diminishing marginal returns from additional data collection. Rather than treating data collection as continuing until a predetermined “saturation point,” we recognise that thematic richness is co-constructed through researcher interpretation and that additional interviews would always yield some new nuances (36). The decision to stop reflected a pragmatic judgement that the dataset supported robust thematic development and meaningful integration with quantitative findings.

FGDs are most effective when group size remains manageable (6–12 participants per session), allowing for in-depth discussion and equal participation (37). The focus group enabled exploration of shared workplace meanings and collective experiences. Individual interviews supported detailed accounts of sensitive topics that might be constrained by social desirability in group settings (financial strain, mental health stigma, discrimination experiences). This approach allowed participants to co-construct shared meaning in the FGD, then elaborate on personal experiences in confidential interview space. This sample size aligns with recent findings that a sample of 15–23 participants typically achieves near saturation in qualitative data collection, indicating minimal benefit in collecting additional data beyond this point (38). Table 1 provides the demographic profile of qualitative participants, demonstrating diversity across race, gender, age and qualification.

**Table 1:** Demographic and professional profile of participants (qualitative).

Participant	Race	Age	Qualification	Gender
1	Mixed ancestry	42	Masters	Female
2	Black	36	Masters	Female
3	Black	53	Bachelors	Male
4	Black	42	Bachelors	Female
5	White	44	Masters	Male
6	White	61	Matric	Female
7	Black	42	Bachelors	Female
8	Indian/Asian	38	Bachelors	Female
9	Black	36	Diploma	Female
10	Indian/Asian	40	Diploma	Female
11	Indian/Asian	55	Diploma	Male
12	White	50	Masters	Male
13	Black	41	Diploma	Female
14	Indian/Asian	46	Bachelors	Female
15	Indian/Asian	43	Bachelors	Female
16	Mixed ancestry	53	Diploma	Female
17	White	61	Diploma	Female
18	White	47	Bachelors	Male
19	Black	57	Diploma	Male

### Measures

All quantitative measures were selected based on established psychometric properties, prior use in South African samples where available, and conceptual alignment with the study’s well-being framework (Figure 1, micro level). Measures assessed five well-being outcomes.

### Mental well-being

The 14-item Warwick–Edinburgh Mental Well-Being Scale (WEMWBS) measures positive mental health capturing both hedonic and eudaimonic aspects of well-being (39). Items are rated on a 5-point Likert scale (1 = none of the time to 5 = all of the time), with total scores ranging from 14 to 70. Higher scores indicate higher mental well-being. WEMWBS demonstrates strong psychometric properties across diverse settings (40) and has been validated for use in South Africa (41).

### Perceived stress

The 4-item Perceived Stress Scale (PSS-4) assesses the degree to which situations are appraised as stressful (42, 43). Items are rated on a 5-point Likert scale (0 = never to 4 = very often), with total scores ranging from 0 to 16. Higher scores reflect higher perceived stress. A score of 6 or above has been used as a threshold indicating comparatively high perceived stress (43). The PSS-4 has been used in South African research (44).

### Work engagement

The 3-item Utrecht Work Engagement Scale (UWES-3) captures vigour, dedication, and absorption (45). Items are rated on a 7-point Likert scale (0 = never to 6 = always), with higher scores indicating higher engagement. The UWES-3 has demonstrated sound psychometric properties (46) and has been validated in South African samples (47).

### Life satisfaction

The 5-item Satisfaction with Life Scale (SWLS) measures the cognitive component of subjective well-being (48). Items are rated on a 7-point Likert scale (1 = strongly disagree to 7 = strongly agree), with total scores ranging from 5 to 35. Higher scores indicate greater life satisfaction. The SWLS demonstrates high internal consistency and has been applied in South African research (49).

### Flourishing

The 8-item Flourishing Scale provides a summary score representing self-perceived success across key domains of psychological well-being and social functioning (50). Items are rated on a 7-point Likert scale (1 = strongly disagree to 7 = strongly agree), with total scores ranging from 8 to 56. Higher scores indicate higher flourishing. The scale has demonstrated strong psychometric properties and has been used in South African studies (51).

### Demographic variables

Demographic variables included gender (male, female), race (Black African, White, Indian/Asian, Mixed ancestry), age cohort (Millennials, Generation X, Generation Z and Baby Boomers), income category (low, medium, high), and education level (Matric, Diploma, Bachelor’s, Master’s). These variables were used to examine patterned differences in workplace well-being outcomes.

Consistent with intersectionality theory, demographic categories were treated not as essential individual attributes but as markers of social positioning and structural context (17). Gender differences in well-being reflect gendered role expectations and structural burdens; income differences reflect economic security and culturally mediated obligations. This conceptual framing informed both quantitative interpretation and qualitative inquiry.

### Data collection procedures

#### Qualitative data collection

The FGD and interviews were conducted at the organisation’s office in private meeting rooms. After careful consideration of methodological trade-offs, qualitative sessions were not audio-recorded. This decision was deliberate and theoretically informed rather than a limitation, reflecting three intersecting ethical and epistemological priorities.

First, the organisational context created power asymmetries that recording would have amplified. The lead researcher held a managerial position within the organisation at the time of data collection, creating an inherent insider-researcher dynamic. Although insider status conferred advantages (established trust, contextual knowledge, genuine commitment to employee well-being as Human Resource manager), it also introduced risks of perceived surveillance (52). Employees may have feared that recorded statements criticising workplace conditions, disclosing mental health struggles, or describing discrimination could be used against them in performance evaluations or disciplinary processes, despite explicit confidentiality assurances (52). The presence of recording devices would have materially signalled institutional documentation, heightening these fears and likely constraining candour (53).

Second, the study’s focus on intersectional workplace inequities in post-apartheid South Africa engaged topics that participants explicitly identified as taboo, stigmatised, or professionally risky to discuss on record. In preliminary conversations, multiple employees expressed concern that:

Black employees feared that discussing historical racial inequities (apartheid legacies, ongoing discrimination, racialized financial burdens like Black tax) might be perceived as “playing the race card” or making White colleagues uncomfortable, potentially damaging workplace relationships or being labelled as “difficult.” Post-apartheid South Africa is characterised by complex racial silences in workplace settings, where structural inequalities persist but explicit discussion is often avoided to maintain surface-level harmony.

Black men feared that disclosing mental health struggles, caregiving stress, or emotional vulnerability would violate cultural masculinity norms, leading to being perceived as weak, unreliable, or unfit for leadership. South African masculine ideals emphasise stoicism, provision, and emotional restraint, with mental health disclosure heavily stigmatised (28). Recording these admissions would have created permanent evidence of “weakness,” intensifying reluctance to speak honestly.

Women, particularly single mothers and Black/Mixed ancestry women managing dual burdens, worried that describing work-life conflict, caregiving exhaustion, or financial strain would signal reduced commitment or competence, potentially affecting future performance evaluations, promotion opportunities, or managers’ perceptions of their reliability. The ideal worker norm assumes unencumbered availability; disclosing caregiving constraints risks being categorised as a “liability” rather than an asset.

Employees across demographics expressed concern that recorded criticism of workplace practices (inadequate mental health support, insufficient flexibility, workload inequities) could be traced back to them despite anonymization promises, creating reputational or career risks.

These fears are not paranoia but rational assessments of risk in contexts where workplace hierarchies, historical power relations, and gender/race/class inequalities intersect. Recording would have fundamentally altered the data generated: participants would have self-censored, offered socially desirable responses, or declined participation altogether, undermining the study’s capacity to surface authentic experiences of intersectional marginalisation.

Third, building psychological safety required visible commitments to confidentiality that non-recording materially demonstrated. Rather than simply stating “your responses are confidential,” the absence of recording devices served as a concrete symbol that no permanent institutional record was being created. Throughout sessions, the researcher explicitly and repeatedly reassured participants that their words would not be quoted verbatim or attributed to them by name, and that the purpose was to understand collective patterns rather than individual disclosures. This approach aligns with feminist and critical methodologies that prioritise participant protection and relational ethics over methodological “purity” (54).

The following structured protocol was used to preserve data quality:

During sessions, the lead researcher took detailed longhand notes capturing: (a) participant statements in as close to verbatim form as possible, with quotation marks used in notes to denote exact phrasing captured in the moment; (b) paraphrased summaries for longer responses; (c) non-verbal cues including pauses, emotional reactions, and silences; and (d) contextual observations on group dynamics and power relations.

Within 24 h of each session, notes were expanded into fuller narrative accounts. This short window was deliberate: it minimised recall decay and allowed the researcher to reconstruct dialogue while memory remained fresh. Expanded notes included recalled verbatim quotes, thematic impressions, analytic memos, and reflexive commentary on researcher positioning.

To verify accuracy, the second researcher reviewed a sample of expanded notes and provided feedback on detail sufficiency, internal consistency, and potential interpretive bias. Where reconstructed quotes appeared uncertain, they were either paraphrased or removed from the final analysis. This process directly mitigated recall bias by introducing an independent check on the lead researcher’s reconstructions.

Quotes presented in this manuscript represent the researchers’ best reconstruction of participant language. Shorter quotes of one to two sentences are likely near-verbatim, captured in the moment with quotation marks in the original notes. Longer passages reflect paraphrased content preserving meaning and tone. We claim fidelity to participants’ intended meanings rather than perfect verbatim accuracy.

This approach prioritised participant safety and data authenticity over conventional recording procedures. In this specific organisational context, non-recording was not a methodological compromise but a condition for obtaining honest accounts from employees discussing sensitive racial, financial, and mental health experiences. The structured note-taking and expansion protocol described above ensured analytic rigour despite the absence of recordings.

Comparison to alternative approaches. We considered several alternatives: (1) recording with option to delete sections, but this still required initial disclosure to a device; (2) anonymous written surveys, but these lack conversational depth; (3) external (non-managerial) interviewer, but this would sacrifice insider contextual knowledge and established trust. Each alternative involved different trade-offs. Our approach optimised for psychological safety and depth of critical disclosure within the constraints of an insider-researcher design.

### Data analysis

#### Quantitative analysis

Quantitative analyses were conducted using SPSS. Descriptive statistics (frequencies, medians, interquartile ranges) were calculated for all demographic variables and well-being outcomes. Shapiro–Wilk tests assessed normality of distribution for all continuous variables. Results indicated that none of the well-being constructs met assumptions of normality (all *p* < 0.05); therefore, non-parametric methods were used for hypothesis testing (55).

Two-group comparisons (gender) were examined using Mann–Whitney U tests. Comparisons involving more than two categories (generation, income, education, race) were examined using Kruskal–Wallis H tests (56, 57). Statistical significance was set at *α* = 0.05. Findings are interpreted cautiously and positioned as hypothesis-generating rather than confirmatory.

#### Multiple testing considerations

Given the exploratory nature of this study, we conducted 25 statistical tests (5 demographic predictors × 5 well-being outcomes) without formal correction for multiple comparisons. Applying a conservative Bonferroni correction (α = 0.05/25 = 0.002) would retain only the income–perceived stress association (*p* < 0.001) as statistically significant, while a less conservative false discovery rate (FDR) correction would retain income’s effects on mental well-being (*p* = 0.033), life satisfaction (*p* = 0.011), and perceived stress (*p* < 0.001), as well as gender’s effect on mental well-being (*p* = 0.015). However, given the study’s hypothesis-generating aims, single-organisation context, and emphasis on mixed-methods integration rather than confirmatory hypothesis testing, we report findings at the conventional α = 0.05 threshold while acknowledging that some associations may represent Type I errors. The qualitative phase was designed to evaluate the plausibility of quantitative patterns by examining whether lived experiences align with statistical signals, providing a form of triangulation that strengthens confidence in replicated patterns (e.g., women’s caregiving burdens explaining the gender–mental well-being association) while contextualising potentially spurious findings.

#### Qualitative analysis

Qualitative data were analysed using reflexive thematic analysis following the six-phase approach: familiarisation with data, generating initial codes, constructing themes, reviewing and refining themes, defining and naming themes, and producing the report (58). Reflexive thematic analysis was selected because it supports systematic identification of patterned meaning while acknowledging the interpretive role of the researcher (58).

To enhance trustworthiness, the following strategies were employed: prolonged engagement with participants, building rapport and trust; triangulation of data sources (FGD, individual interviews, survey); thick description using detailed field notes and expanded narrative accounts; reflexive memoing documenting analytic decisions and researcher assumptions; informal member checking during interviews where the researcher summarised participant statements and invited clarification; and peer debriefing with academic supervisors and HR colleagues to interrogate emerging interpretations (59). These strategies address the three core trustworthiness criteria: credibility (through prolonged engagement, triangulation, and member checking), dependability (through reflexive memoing and peer debriefing), and confirmability (through thick description and transparent reporting of analytic decisions).

#### Intersectionality analytic strategy

Intersectionality theory informed both conceptual framing and analytic approach. Given the sample size (*n* = 87), statistical power was insufficient to model higher-order interactions (e.g., gender × race × income). Quantitative analyses therefore examined main effects of demographic categories as an initial mapping step to identify which axes show measurable patterning in well-being outcomes. Modelling intersectional interactions would have required either large samples enabling three-way interaction terms, or sufficient cell sizes for meaningful subgroup comparisons (e.g., comparing Black women caregivers to White women caregivers). With *n* = 87, four racial categories, two genders, and three income levels, such analyses were not statistically feasible.

Consistent with intersectionality theory (17), the most meaningful inequities may occur at specific intersections (e.g., Black women with caregiving roles; Black professionals carrying kin obligations) rather than as uniform main effects. The qualitative phase was explicitly designed to capture these intersectional mechanisms by exploring how multiple identities simultaneously shape workplace experiences. This two-phase approach prioritises depth of understanding over statistical precision, positioning the study as exploratory and hypothesis-generating. Mixed-methods integration then triangulates quantitative patterns with qualitative mechanisms to explain how and why disparities emerge through intersectional processes—an approach aligned with pragmatic intersectionality frameworks that emphasise surfacing mechanisms over quantitative interaction terms.

#### Researcher positionality and reflexivity

Reflexivity is essential in interpretive qualitative research because meaning is co-constructed between researcher and participants (58). The research team comprised two researchers whose distinct positionalities strengthened analytic rigour through complementary perspectives.

The lead researcher is a South Asian male HR leader with multiple years of managerial experience in Asia and Africa. At the time of data collection, he was employed as a manager within the organisation. This insider position conferred both advantages and challenges. Advantages included established rapport and trust, deep contextual knowledge of organisational dynamics, and credibility as someone genuinely invested in employee well-being. Challenges included potential power dynamics (participants may have been reluctant to criticise job demands) and risk of interpretive bias shaped by prior intervention assumptions and managerial perspective.

The second researcher is an African woman with specialisation in industrial psychology and multiple years of academic research experience. Her outsider position relative to the organisation, combined with academic expertise in workplace well-being and African contexts, provided critical distance and theoretical grounding. Her positionality as an African woman researcher also enabled culturally attuned interpretation of gendered and racialized workplace experiences, particularly regarding intersectional mechanisms that the lead researcher’s positioning might not fully capture.

To mitigate potential bias and enhance transparency, reflexive strategies were employed throughout: reflexive memoing during data collection and analysis documenting assumptions and analytic decisions, peer debriefing between the two researchers to interrogate emerging themes and challenge interpretations, bracketing of prior expectations to remain open to disconfirming evidence, and thick description in reporting using detailed quotations to allow readers to assess interpretive claims. The complementary insider-outsider, practitioner-academic, and gendered-racialized positionings of the research team strengthened the study’s capacity to surface both organisational realities and broader structural dynamics shaping workplace well-being in post-apartheid South Africa.

Despite these strategies, the study’s findings remain shaped by the researchers’ interpretive lenses. Readers are invited to consider how alternative positionings might yield different insights.

### Ethical considerations

Ethical approval to conduct this study was obtained from the University of Johannesburg Department of Industrial Psychology and People Management Research Ethics Committee (No. IPPM-2022-618[D]). Participation was voluntary. Participants provided informed consent electronically after reviewing an information sheet. Survey responses were anonymous with no identifying information collected. Qualitative data were de-identified during transcription of field notes, with participants assigned pseudonyms. All identifying details were removed or generalised. Data were stored securely in password-protected files accessible only to the research team.

### Methodological limitations

Several methodological limitations warrant acknowledgement. First, the single-organisation design constrains external validity. Findings reflect one healthcare sector organisation in Johannesburg and cannot be generalised to other sectors, organisational sizes, or regions of South Africa. The study is explicitly positioned as exploratory and hypothesis-generating, establishing plausible mechanisms for investigation in larger, multi-site samples rather than producing population-level estimates. Researchers seeking to build on these findings should prioritise multi-site replication with sufficient sample sizes to model interaction effects.

Second, qualitative data were captured through field notes rather than audio recordings. As detailed in the Data Collection Procedures section, this decision was deliberate and ethically justified given the insider-researcher context and the sensitivity of topics discussed. A structured note-taking and expansion protocol was employed to preserve data quality, and reconstructed quotes were independently reviewed by the second researcher. Nevertheless, verbatim precision cannot be guaranteed, and this remains a limitation of the qualitative component.

Third, the cross-sectional design limits causal inference. The study identifies associations and plausible mechanisms but cannot establish directionality. For example, it is not possible to determine from this data whether Black tax causes financial strain that reduces well-being, or whether employees with lower well-being perceive Black tax as more burdensome. Longitudinal designs are needed to examine causal pathways.

Fourth, the insider-researcher dynamic, while managed through reflexive strategies, may have influenced participant responses. Despite non-recording and confidentiality assurances, some participants may have self-censored when discussing workplace criticism or personal financial strain.

## Result

### Quantitative findings

#### Demographic associations and main effects

Across all demographic comparisons (5 demographic variables × 5 well-being outcomes = 25 tests), four statistically significant associations emerged, while 21 tests yielded non-significant results (Table 2). This pattern is itself informative: the absence of widespread main effects suggests that well-being disparities may operate through intersectional mechanisms rather than uniform demographic differences.

**Table 2:** Hypothesis testing results (author’s own construction).

Construct	Demographic	Statistic	*p*-value	Test	Significant association
WEMWBS	Gender	500.000	0.015	Mann–Whitney U	Yes
WEMWBS	Generation	726.000	0.061	Mann–Whitney U	No
WEMWBS	Income	6.821	0.033	Kruskal-Wallis	Yes
WEMWBS	Education	0.828	0.661	Kruskal-Wallis	No
WEMWBS	Race	0.176	0.981	Kruskal-Wallis	No
SWLS	Gender	674.500	0.438	Mann–Whitney U	No
SWLS	Generation	844.000	0.386	Mann–Whitney U	No
SWLS	Income	8.984	0.011	Kruskal-Wallis	Yes
SWLS	Education	4.246	0.120	Kruskal-Wallis	No
SWLS	Race	0.738	0.864	Kruskal-Wallis	No
Flourish	Gender	677.500	0.454	Mann–Whitney U	No
Flourish	Generation	807.000	0.236	Mann–Whitney U	No
Flourish	Income	5.251	0.072	Kruskal-Wallis	No
Flourish	Education	1.558	0.459	Kruskal-Wallis	No
Flourish	Race	0.617	0.893	Kruskal-Wallis	No
UWES	Gender	682.000	0.477	Mann–Whitney U	No
UWES	Generation	788.000	0.175	Mann–Whitney U	No
UWES	Income	3.270	0.195	Kruskal-Wallis	No
UWES	Education	5.106	0.078	Kruskal-Wallis	No
UWES	Race	0.519	0.915	Kruskal-Wallis	No
PSS	Gender	570.500	0.075	Mann–Whitney U	No
PSS	Generation	808.500	0.237	Mann–Whitney U	No
PSS	Income	15.656	<0.001	Kruskal-Wallis	Yes
PSS	Education	3.314	0.191	Kruskal-Wallis	No
PSS	Race	2.256	0.521	Kruskal-Wallis	No

Hypothesis testing results: Associations between demographic variables and workplace well-being outcomes (*n* = 87).

The four significant associations were:

Gender → Mental well-being (WEMWBS): Male employees reported significantly higher mental well-being than female employees (U = 500.000, *p* = 0.015, *r* = 0.261), indicating a small-to-moderate effect.

Income → Mental well-being (WEMWBS): Income significantly influenced mental well-being (H = 6.821, *p* = 0.033, ε^2^ = 0.057), with higher-income employees reporting better mental health outcomes. Dunn–Bonferroni post-hoc comparisons did not identify statistically significant pairwise differences between specific income groups after adjustment.

Income → Life satisfaction (SWLS): Income significantly influenced life satisfaction (H = 8.984, *p* = 0.011, ε^2^ = 0.083), with higher-income employees reporting greater satisfaction with life overall. Dunn–Bonferroni post-hoc comparisons indicated a significant difference between the below-average and above-average income groups (*p* = 0.028). This comparison should be interpreted cautiously given the small size of the below-average income group (*n* = 5).

Income → Perceived stress (PSS): Income significantly influenced perceived stress (H = 15.656, *p* < 0.001, ε^2^ = 0.163), with higher-income employees reporting lower stress. This was the strongest association in the dataset. Dunn–Bonferroni post-hoc comparisons indicated a significant difference between the average and above-average income groups (*p* < 0.001).

No significant main effects were observed for generation or race across any well-being construct. Additionally, gender did not significantly predict life satisfaction, flourishing, work engagement, or perceived stress; income did not predict flourishing or work engagement; and education did not predict any well-being outcome (Table 2).

#### Interpretation of quantitative patterns

Three key observations emerged from this pattern of findings, each requiring qualitative elaboration to understand the underlying mechanisms.

First, gender emerged as a significant predictor of mental well-being specifically, but not other well-being dimensions. This finding aligns with international evidence demonstrating persistent gender gaps in subjective well-being (11, 31). However, the selectivity of this finding is notable: gender did not predict stress, engagement, life satisfaction, or flourishing. The mechanism underlying this disparity requires qualitative elaboration: why do women report lower mental well-being in this organisational context, and through what pathways does gender shape daily workplace experiences?

Second, income emerged as the most powerful and consistent predictor of workplace well-being, significantly predicting mental well-being, life satisfaction, and perceived stress. However, income did not predict flourishing or work engagement, suggesting that eudaimonic dimensions of well-being (meaning, purpose, work passion) may be independent of financial resources.

Importantly, income’s effects may be culturally mediated rather than universal. The qualitative component explores whether and how kin-based financial transfers may help explain these relationships, potentially constraining income’s protective effects for Black professionals specifically.

Third, the absence of significant main effects for race and generation does not indicate the absence of racialized or generational dynamics. Intersectionality theory cautions against interpreting null main effects as evidence that social categories are irrelevant (17). Instead, race and generation may operate most powerfully at specific intersections rather than as uniform effects across all employees. For example, the experiences of Black women who are single mothers may differ substantially from both Black men and White women, but this difference may not be detectable as a race main effect in a sample of 87. Similarly, younger Black professionals managing both career mobility and kin obligations may experience distinct stressors not captured by generation main effects. The qualitative findings support this interpretation by surfacing how race intersects with gender, caregiving roles, and financial obligations to shape workplace well-being at specific meso-level positions (Figure 1).

These quantitative patterns establish that workplace well-being is patterned by social positioning in this South African organisational context, while simultaneously demonstrating the limitations of main-effects analyses for capturing intersectional mechanisms. The qualitative analysis that follows explains these patterns by exploring the lived experiences through which macro-level social determinants translate into meso-level intersectional positioning and micro-level workplace demands and resources.

### Qualitative findings

The qualitative analysis identified four intersecting themes that explain quantitative patterns and align with the theoretical model (Figure 1). Each theme demonstrates how macro-level social determinants shape meso-level intersectional positions, which translate into micro-level job demands and resources, ultimately influencing well-being outcomes.

#### Theme 1: “back at the office, I could breathe again”

Quantitative results showed that gender significantly influenced mental well-being (*p* = 0.015), with women reporting lower WEMWBS scores than men. Qualitative accounts clarified the mechanisms. Women consistently described the post-pandemic return to office as a partial reprieve from intensified domestic load. Rather than representing a simple return to normal, the workplace was framed as a space of relative psychological relief where women could temporarily step out of caregiving intensity and experience brief autonomy.

*I was so relieved when we were asked to come back to the office after the pandemic. This is the space where I can breathe and have some time for myself. I can make myself a cup of coffee and have it in peace.* (Participant 16)

Women described navigating a persistent cognitive split between home and work, with emotional labour extending across both domains:

*When I am at work, I am thinking of my children. When I am at home, I am worried about job security.* (Participant 10)

Several participants articulated the second shift in concrete terms, describing exhaustion produced by cumulative caregiving, domestic labour, and professional performance expectations:

*I wake up at 4 AM to prepare everyone's lunch, get the children ready for school, then I rush to work where I must be fully present. After work, there's homework, dinner, and household management. When do I rest? When do I take care of my own well-being?* (Participant 8)

These narratives suggest that women’s lower mental well-being scores were not attributable to individual coping deficits but were rooted in unequal distributions of unpaid labour and recovery time.

Alignment with framework (Figure 1). At the macro level, patriarchal cultural systems structure gendered divisions of labour. At the meso level, women’s intersectional positioning (gender plus caregiving role expectations) amplifies micro-level job demands (role conflict, time pressure, cognitive load), thereby eroding mental well-being. Women experience high demands without proportional access to the resource of recovery time, creating a sustained demands–resources imbalance.

#### Theme 2: not all women are positioned the same

Qualitative findings demonstrated that women did not represent a uniform category. Participants emphasised that women’s experiences were shaped by intersectional positioning, particularly the interaction of gender, race, and marital status. White women acknowledged that although they experienced dual burdens, Black and Mixed ancestry women often carried sharper strain due to cultural expectations, economic precarity, and family surveillance.

*I can be an independent Black woman who has achieved a higher education and become a team manager at work. But when I visit my parents' home, I have to leave these at my doorstep. For my father, he still needs me to do cooking, cleaning and conform to the traditional role of women. Sometimes I think I live two lives.* (Participant 4)

*As women, we are constantly juggling multiple responsibilities, caring for the family, managing the household, and fulfilling church duties, on top of our jobs. While men are still mostly expected to focus only on work.* (Participant 2)

Participants described the structural and generational dimensions of caregiving, noting that the burden often shifts to grandmothers and older women in contexts of single-parent households:

*If you look at the data, it shows that over 60% of South African children are raised in single-parent households. And who's taking care of them? It's the grandmothers or the gogos.* (Participant 13)

One participant explicitly named the complexity of navigating multiple racial and gendered identities:

*White and black women in South Africa have distinct identities. It is tough and blurred for mixed ancestry women.* (Participant 16)

Alignment with framework (Figure 1). This theme helps explain why race did not show significant main effects in the quantitative results. The most consequential differences appeared not between racial categories in aggregate but within gendered and familial intersections. At the macro level, historical legacy (apartheid) and cultural systems (patriarchal norms, collective obligations) shape who carries which burdens. At the meso level, these determinants converge in specific intersectional positions (Black woman plus caregiver plus professional), which intensify micro-level demands (code-switching, cultural surveillance, extended-family responsibilities) and reduce available resources.

#### Theme 3: black tax

Income emerged as the strongest quantitative predictor of well-being (Table 2), with higher income associated with better mental health, life satisfaction, and lower stress. However, qualitative narratives revealed that income’s protective effects were uneven and mediated by culturally embedded obligations, particularly among Black professionals.

*We have the Black tax to pay. In my income, I need to handle not just my family expenses, but the whole community whenever they need. Many in my community supported us when my parents struggled; now they expect me to support them. This can be a so draining and hand-to-mouth situation.* (Participant 3)

*Sometimes I have to choose between saving for my own children's education and helping my cousin pay for her medical bills. There's always someone who needs help, and you can't say no because they helped your family when you had nothing.* (Participant 19)

Participants described financial strain as a social and psychological constraint, rather than merely a budgeting issue. Some reported needing to conceal their financial progress to avoid social pressure or conflict:

*I have to downplay my income and lifestyle. I have to tell my children not to show their fancy toys, tell my partner not to put pictures of our holidays on Facebook.* (Participant 2)

Alignment with framework (Figure 1). These accounts indicate that financial stress cannot be interpreted purely through salary level. Although income’s protective effects were strong and consistent quantitatively (predicting mental well-being, life satisfaction, and perceived stress), qualitative findings revealed that these benefits are culturally mediated. At the macro level, economic structure (apartheid-era wealth exclusion, intergenerational inequality) and cultural systems (ubuntu reciprocity, collective obligation) create the conditions for Black tax. At the meso level, Black professionals occupy a specific intersectional position (upward mobility plus kin obligation) that mediates how income translates into well-being. At the micro level, this positioning manifests as ongoing financial pressure (a job demand), which constrains income’s protective effects: higher salary does not guarantee proportional psychological benefit when resources are redistributed through kin obligations. This explains why, despite income’s significant quantitative effects across multiple well-being domains, Black professionals described persistent financial strain even with professional employment.

#### Theme 4: be a man

Although the survey suggested men had higher mental well-being scores than women, qualitative findings demonstrated that men’s well-being was constrained by restrictive masculine norms. Participants described a narrow emotional script emphasising endurance, provision, and silence. This was especially pronounced among Black men, where help-seeking was described as culturally stigmatised.

*In our society, it is very difficult to talk about our (men's) mental health. We have been taught to be tough and ensure. Otherwise, we are labelled as weak.* (Participant 18)

*Men are expected to provide. If we are weak, we are good for nothing.* (Participant 5)

Some men linked emotional distance to intergenerational patterns of masculinity:

*I never had my father show any affection to me while growing up. On the other hand, he treated my younger sister like a doll.* (Participant 11)

Men also described hidden caregiving and provider stress that remained unspoken, particularly during family illness or crisis:

*My wife went through cancer. I have to take care of the whole family, but I can't ask for support from anyone. My wife was always my support, but today, due to cancer, I don't want to burden her further.* (Participant 12)

Alignment with framework (Figure 1). These narratives support the interpretation that men’s distress may be under-reported in survey measures due to stigma and emotional suppression. At the macro level, cultural systems (patriarchal norms, masculine ideals) define acceptable male emotional expression. At the meso level, men are positioned as providers and stoics, which constrains access to the micro-level resource of emotional support and help-seeking. This provides a plausible explanation for why gender differences were significant for mental well-being but not for perceived stress: men may experience stress but suppress its expression and disclosure, masking distress in self-report measures. Recent South African research has similarly described culturally shaped dissonance in men’s mental health expression (28).

### Mixed-methods integration

The integration section does not restate findings from each strand but draws meta-inferences — conclusions that neither quantitative nor qualitative data could support alone. Table 3 presents a joint display summarising how each quantitative pattern maps onto a qualitative mechanism, the intersectional process connecting them, and the equity implication that follows. Three illustrative examples are elaborated below.

**Table 3:** Joint display: integrating quantitative patterns, qualitative mechanisms, and theoretical framework.

Quantitative pattern	Subgroup variations (qualitative)	Intersectional mechanism	Framework level	Equity implication
Gender → Mental Well-Being (*p* = 0.015), Women report lower WEMWBS	Black/Mixed ancestry women carry sharper strain than White women	Gendered unpaid labour × racial/cultural caregiving expectations- Second shift amplified by extended-family obligations and cultural surveillance	Macro: Patriarchal norms	Caregiver-sensitive leave policies; subsidised childcare; workload audits disaggregated by gender × race
	Grandmothers bear caregiving for single-parent households		Meso: Caregiving role × race × marital status	
	“Living two lives” (professional vs. traditional daughter/wife roles)		Micro: Time poverty, role conflict	
Income → Mental Well-Being, Life Satisfaction, Perceived Stress (*p* = 0.033, 0.011, <0.001), Higher income = better outcomes	Black professionals experience persistent financial strain (despite stable income)	Black tax mediates income’s protective effects- Kin-based financial obligations redistribute resources, constraining psychological benefits for Black employees	Macro: Apartheid wealth exclusion, ubuntu reciprocity	Financial wellness programmes acknowledging Black tax; emergency assistance
	White employees’ income translates more directly to security		Meso: Black professional × upward mobility × kin obligation	
	Scared to flaunt income due to societal pressures		Micro: Financial pressure despite high salary	
No Race Main Effects, Race does not predict well-being in aggregate	Black women navigate professional/traditional identity splits	Race operates through intersections, not uniformly- Race × gender, race × income, race × masculine norms create distinct experiences at specific positions	Macro: Historical inequality, cultural systems	Targeted support for specific vulnerable groups (black/mixed ancestry single mothers)
	Mixed ancestry women experience ambiguous positioning		Meso: Race intersects with gender, income, caregiving	
	Black men face provider expectations + constrained help-seeking		Micro: Code-switching demands, cultural surveillance	
No Gender → Perceived Stress (*p* = 0.075), Men/women report similar stress levels	Men describe hidden provider stress, illness caregiving	Masculine norm constraints suppress stress disclosure- Men (especially black men) experience stress/mental health concerns but do not report it due to stigma	Macro: Patriarchal masculine ideals	Anonymous EAP access; normalise vulnerability through leadership modelling; peer support groups
	Cultural norms for men: “weak” to seek help		Meso: Male × provider role × emotional stoicism	
	Emotional suppression masks distress		Micro: Help-seeking as unavailable resource	

First, gender differences in mental well-being are driven by structural caregiving burdens, not individual differences. Quantitative analyses identified gender as a significant predictor of mental well-being (*p* = 0.015), with women reporting lower WEMWBS scores than men. Qualitative findings clarified that this disparity is rooted in unequal distributions of unpaid labour, recovery time, and role conflict. To illustrate the integration directly: the quantitative signal that women score lower on WEMWBS prompted the qualitative inquiry to explore why. Participants described waking at 4 a.m. to manage household and childcare responsibilities before arriving at work expected to be fully present—a pattern that explains lower positive mental health scores without requiring any assumption of individual psychological deficit.

Women’s meso-level positioning (caregiving roles, cultural surveillance) amplifies micro-level job demands (time pressure, cognitive load), creating a sustained demands–resources imbalance. This gendered pattern was selective—gender did not predict stress, life satisfaction, engagement, or flourishing—suggesting that the disparity operates through specific pathways affecting positive mental health rather than generalised distress.

Second, income emerged as the most powerful and consistent structural determinant of workplace well-being, yet its protective effects are culturally mediated through Black tax.

Income significantly predicted mental well-being, life satisfaction, and perceived stress (Table 2), establishing financial security as a broad protective factor. However, qualitative accounts revealed that income’s protective effects are not uniform across racial groups. Black tax—the culturally embedded expectation to financially support extended family and community members—mediates the relationship between salary and well-being. Black professionals described ongoing financial strain despite stable employment, having to choose between saving for their own children’s education and helping extended family members, and needing to conceal financial progress to avoid social pressure. This creates a paradox: although higher income should reduce stress and enhance well-being (as demonstrated quantitatively), kin-based financial obligations redistribute resources such that salary parity does not guarantee psychological benefit parity.

This finding challenges simplistic assumptions about income as a universal protective factor and underscores the importance of examining how macro-level economic inequality and cultural systems (apartheid-era wealth exclusion, ubuntu reciprocity) shape meso-level obligations (kin financial transfers), which then sustain micro-level financial pressure despite professional employment. Black tax effectively operates as an additional job demand that constrains the psychological benefits of higher income for Black employees specifically.

Third, the absence of race-based main effects reflects intersectional mechanisms operating beneath aggregate comparisons. Race did not show significant main effects in quantitative analyses across any well-being construct. Rather than indicating the absence of racialized dynamics, qualitative findings demonstrated that race operates most powerfully through intersectional mechanisms rather than uniform effects. The integration insight here is methodological as much as substantive: In this study, the small-sample quantitative design was not well positioned to detect racialized disparities that appeared to operate through intersecting positions rather than as uniform main effects. This has direct implications for how workplace equity research is designed. Black women described navigating two lives (professional identity at work, traditional gender roles at home), Black professionals carried Black tax obligations regardless of their income level, and mixed ancestry women described navigating ambiguous racial positioning. These race-specific stressors do not appear as race main effects in small organisational samples when analysed through demographic categories, but they remain influential through lived experience and culturally shaped stress pathways.

This interpretation aligns with intersectionality theory’s core proposition that inequities emerge through interacting social positions rather than isolated demographic categories (17). For example, the finding that income predicts mental well-being quantitatively, but Black tax constrains this protective effect qualitatively, demonstrates how race mediates income’s influence without producing a detectable race main effect. The intersectional mechanisms are evident: race × income interactions shape financial strain, race × gender interactions shape caregiving burdens, and race × masculine norms interactions shape help-seeking constraints.

Joint display demonstrating meta-inferences linking quantitative demographic patterns to qualitative intersectional mechanisms, theoretical framework levels (Figure 1), and equity-focused intervention implications. This table illustrates how main-effects analyses (quantitative) combined with intersectional inquiry (qualitative) enable mechanistic understanding that neither method alone could provide.

## Discussion

### Overview of key findings

This study examined workplace well-being through an intersectional social determinants lens in a South African organisational context using an explanatory sequential mixed-methods design. The study makes three primary contributions to the underdeveloped Global South workplace mental health literature.

First, quantitative findings demonstrated that workplace well-being is patterned by social positioning, with income emerging as the most powerful structural determinant. Gender significantly predicted mental well-being (*p* = 0.015), with women reporting lower scores than men. These patterns provide initial empirical evidence that well-being disparities exist within South African workplaces and warrant equity-focused attention—details are reported in Table 2.

Second, qualitative findings explained how these disparities are produced through intersectional mechanisms that complicate quantitative patterns. The gendered caregiving and masculine norm findings are consistent with existing South African and international literature (28, 31) and serve primarily to contextualise and confirm known patterns within this organisational setting. Most critically, although income’s protective effects were strong quantitatively, they were mediated by culturally embedded obligations, particularly Black tax, which redistributed financial resources and sustained stress despite salary parity. This is the study’s principal novel finding: it moves beyond confirming that income matters to showing precisely why its effects are uneven across racial groups in post-apartheid workplaces.

Third, mixed-methods integration demonstrated that the Intersectional Social Determinants Model (Figure 1) provides a coherent explanatory framework. The integration revealed that quantitative main effects can obscure culturally specific mechanisms: income should protect well-being, but Black tax mediates this relationship for Black employees specifically.

These findings speak to a growing body of Global South scholarship that challenges the universality of Western well-being frameworks (8, 9). African contexts introduce structural conditions—collective obligations, intergenerational wealth exclusion, culturally enforced gender roles—that individualist resource models were not designed to capture. The present study contributes to this literature by grounding the resource mediation argument in a specific, empirically examined mechanism rather than making a general critique of Western frameworks. More broadly, any context characterised by historical wealth exclusion and culturally enforced kin obligations—including South Asian joint family structures and Latin American remittance economies—presents conditions where income’s protective effects may similarly be socially mediated, suggesting the theoretical argument travels beyond the South African case.

### Gendered dynamics of workplace well-being

Gender emerged as a significant predictor of mental well-being (*p* = 0.015), with men reporting higher WEMWBS scores than women. This finding aligns with international evidence demonstrating persistent gender gaps in subjective well-being (11, 31). South African research similarly indicates that women report lower well-being than men, with caregiving responsibilities contributing to this disparity (27).

The present study’s contribution lies in explaining how this disparity is produced. Qualitative accounts demonstrated that women’s lower mental well-being was anchored in the cumulative second shift of unpaid labour, caregiving, and domestic management.

This finding supports the interpretation that women’s mental well-being is constrained not by individual psychological deficits but by time poverty (60). Time poverty functions as a chronic job demand that depletes energy and erodes mental well-being, while access to recovery time (a critical resource) is unequally distributed by gender.

Alignment with framework (Figure 1): At the macro level, patriarchal cultural systems structure gendered divisions of labour. At the meso level, women’s intersectional positioning amplifies exposure to role conflict and time pressure. At the micro level, this positioning manifests as high job demands without proportional access to resources, creating a sustained demands–resources imbalance that erodes mental well-being (21).

Although gender emerged as a significant predictor quantitatively, qualitative findings demonstrated that women did not represent a uniform category. Black and Mixed ancestry women described carrying sharper caregiving strain than White women due to cultural expectations, economic precarity, and extended-family obligations. One participant described living two lives (performing professional competence at work while conforming to traditional gender roles when visiting family). These narratives illustrate intersectionality theory’s core proposition: that social identities operate simultaneously, producing qualitatively distinct experiences at specific intersections (17).

Although men reported higher mental well-being scores quantitatively, qualitative findings demonstrated that men’s well-being was constrained by restrictive masculine norms. Participants described a narrow emotional script emphasising stoicism, provision, and silence, with mental health disclosure culturally stigmatised as weakness. This finding aligns with South African research demonstrating that cultural perspectives on mental healthcare create significant barriers to men’s help-seeking (28). The present study extends this evidence by suggesting that men’s distress may be under-reported in self-report surveys due to social desirability and internalised stigma. This creates a dual gender pattern in workplace mental health: visible overload among women and hidden strain among men.

### Income, black tax, and culturally mediated financial strain

Income significantly predicted both life satisfaction and perceived stress, supporting the interpretation that financial stability functions as a key social determinant of workplace well-being (18, 30). However, the present study’s contribution lies in demonstrating that income effects are not uniform and must be interpreted through culturally mediated pathways.

Black tax is not simply a financial burden unique to South Africa. It represents a broader theoretical problem for workplace well-being research: when economic resources are subject to socially embedded redistribution obligations, salary level becomes a poor proxy for actual financial security. The Job Demands–Resources model treats income and financial resources as buffers against strain—assets that individuals draw on to manage workplace demands. This assumption holds when resources remain with the individual. It breaks down when cultural and structural conditions systematically redirect those resources elsewhere. Black tax is precisely such a condition. It operates as a relational and socially enforced obligation rooted in apartheid-era wealth exclusion and ubuntu reciprocity, meaning that for Black professionals, a higher salary does not translate proportionally into reduced strain. Income remains protective on average (as the quantitative findings show) but income’s protective benefits may be reduced for those carrying substantial kin obligations. This is not a noise problem solvable by larger samples. It is a conceptual problem: resource frameworks need to account for the social conditions under which resources are retained or redistributed.

Participants described Black tax as an ongoing obligation that reduced personal financial security even among professionals with stable employment. This finding aligns with scholarship describing kin-based transfers as both an expression of ubuntu reciprocity and a constraint on wealth accumulation (14, 15). Some reported monthly financial transfers representing 20–30% of their salary, effectively reducing their economic security to levels below their nominal income bracket.

This finding challenges simplistic assumptions about income as a universal protective factor. Salary parity does not guarantee psychological benefit parity when resources are redistributed through kin obligations. The quantitative finding that income predicts lower stress appears straightforward, but qualitative findings reveal that this relationship operates differently across racial groups. For White employees, higher income likely translates more directly into reduced financial strain and enhanced well-being. For Black employees, higher income provides some protection but is partially offset by Black tax obligations, sustaining financial pressure despite professional employment.

This creates a critical insight: the quantitative income effect represents an average across culturally heterogeneous experiences. The significant *p*-value (*p* < 0.001 for stress) demonstrates that income matters, but qualitative findings reveal that its protective magnitude varies by meso-level intersectional positioning. Black tax effectively operates as an additional micro-level job demand (ongoing financial pressure, kin obligation management, identity concealment) that constrains income’s psychological benefits for Black professionals specifically.

Alignment with framework (Figure 1): At the macro level, economic structure (apartheid-era wealth exclusion, intergenerational inequality) and cultural systems (ubuntu reciprocity, collective obligation) create the conditions for Black tax. At the meso level, Black professionals occupy a specific intersectional position (upward mobility plus kin obligation) that mediates how income translates into well-being. At the micro level, this positioning manifests as ongoing financial pressure, which sustains perceived stress and constrains life satisfaction even among higher-income employees. This multi-level mechanism explains why income’s protective effect, though statistically significant, does not eliminate well-being disparities: the benefits are culturally redistributed.

### Why race did not emerge as a main effect

Race did not show significant main effects in the quantitative analysis across any well-being construct. This finding requires careful interpretation because it could be misinterpreted as indicating that race is irrelevant to workplace well-being in South African contexts.

Intersectionality theory argues that social identities do not operate independently and that inequities often emerge through interacting social positions rather than as uniform main effects (17). In the present study, qualitative findings demonstrated that race operated most powerfully through intersectional mechanisms and mediating pathways rather than uniform effects. Three patterns were particularly evident:

First, qualitative findings suggest that Black tax may condition income’s protective effects for some Black employees. Although income significantly predicted well-being quantitatively, this relationship operates differently across racial groups. This pattern is consistent with a race-conditioned income effect that would not appear as a race main effect in a sample of 87, but is visible in lived experience and may constrain the protective benefits of income for Black professionals.

Second, gendered caregiving burdens intersect with race. Black and Mixed ancestry women described carrying sharper caregiving strain than White women due to cultural expectations (extended-family obligations, ubuntu reciprocity), economic precarity (Black tax reducing available resources for childcare), and cultural surveillance (needing to conform to traditional gender roles when visiting family). These race × gender intersections produce distinct experiences that do not appear as race main effects when analysed through demographic categories.

Third, Mixed ancestry women described navigating ambiguous racial positioning in post-apartheid organisational contexts, experiencing marginalisation within both Black and White employee networks. This specific intersectional experience would not be detectable in analyses comparing broad racial categories.

The absence of significant race main effects in this study is also attributable to sample size and statistical power. The study sample (*n* = 87) was sufficient to detect large main effects but insufficient to model higher-order interactions (e.g., race × gender × income) or conduct meaningful subgroup analyses at multiple intersections. Intersectional effects are often moderate in magnitude because they reflect compounding mechanisms that affect smaller subgroups within the population. For example, if Black tax constrains income’s protective effect specifically for Black employees, this interaction would require adequate statistical power to detect—power that a sample of 87 does not provide.

This finding has important methodological implications. It demonstrates that intersectionality cannot be adequately operationalised through additive demographic comparisons in small samples. Researchers examining workplace well-being equity must employ designs that support detection of intersectional mechanisms, either through large quantitative samples that enable interaction modelling (n > 500), or through qualitative and mixed-methods approaches that surface lived experiences at specific intersections. The present study’s value lies in using mixed methods to demonstrate *how* race operates (through Black tax, gendered caregiving, cultural code-switching) even when race main effects are not statistically detectable.

### Implications for workplace mental health equity

The findings suggest that workplace mental health equity strategies must move beyond universal interventions and address the unequal distribution of demands and resources across employee groups. The Intersectional Social Determinants Model (Figure 1) provides a multi-level framework for organising these implications. In South Africa, these recommendations connect directly to existing policy frameworks including the Employment Equity Act, the Occupational Health and Safety Act, and the National Mental Health Policy Framework and Strategic Plan 2023–2030. These frameworks create legal and institutional scaffolding for equity-oriented workplace interventions, yet implementation at the organisational level remains inconsistent. The present study’s findings suggest that effective implementation requires attending to intersectional burdens rather than applying uniform well-being programmes.

At the macro level, organisations have limited capacity to change structural determinants but can advocate for policy reforms and align internal practices with equity principles. This includes supporting family-friendly labour legislation, advocating for progressive taxation and social protection systems that reduce reliance on informal kin-based transfers, and challenging patriarchal norms through organisational messaging and leadership modelling.

At the meso level, organisations can implement policies that reduce specific burdens experienced by employees at marginalised intersections. For women managing caregiving roles, concrete interventions include flexible start and finish times that accommodate school drop-off and collection, caregiver-sensitive leave provisions that go beyond statutory maternity leave to include emergency family responsibility leave without performance penalty, and subsidised childcare referral services. These directly address the time poverty identified in this study as the primary mechanism constraining women’s mental well-being.

For employees carrying Black tax obligations, generic financial wellness programmes are insufficient. Effective interventions must explicitly acknowledge kin-based financial obligations as a structural reality rather than a personal budgeting problem. This includes confidential financial counselling that incorporates extended family obligations, emergency assistance funds accessible without stigma, and salary benchmarking that accounts for differential cost burdens across racial groups—recognising that nominal salary parity does not produce equivalent financial security.

For men constrained by masculine norms, anonymous access to Employee Assistance Programmes is necessary but not sufficient. Organisational culture change is required: this includes visible leadership modelling of help-seeking behaviour, peer support structures where men can discuss stress without formal disclosure, and manager training that specifically addresses how masculine norms suppress distress reporting and what managers can do to create psychologically safe alternatives.

At the micro level, organisations can modify job demands and resources to improve the demands–resources balance. This includes conducting workload audits to identify chronic overload, establishing clear boundaries around after-hours communication to protect recovery time, enhancing workplace social support through manager training and peer mentoring, increasing autonomy by involving employees in decision-making, and delivering evidence-based well-being interventions through trained HR professionals.

Importantly, organisations should conduct routine equity audits of well-being outcomes, workload distribution, flexibility access, and promotion pathways, disaggregated by intersecting identities (e.g., Black women, White men, younger Black professionals). Rather than assuming uniform benefit from well-being initiatives, equity-focused evaluation should assess differential uptake and outcomes. For example, if financial wellness programmes do not acknowledge Black tax, they may be underutilised by the employees who need them most.

Positioning as exploratory pilot research

This study should be interpreted as exploratory, hypothesis-generating pilot research. The single-organisation setting (*n* = 87) provided contextual depth but constrains generalisability; findings are proof-of-concept rather than population-level estimates.

The study’s value lies not in definitive quantitative estimates but in: (1) surfacing mechanisms through which structural inequalities translate into workplace well-being disparities, (2) developing a conceptually integrated multi-level framework that can guide future research, and (3) generating specific hypotheses that warrant testing in larger, more representative samples.

### Future research directions

Five research directions warrant priority:

First, multi-site replication with larger samples (n > 500) would enable examination of three-way interactions (e.g., race × gender × income) and meaningful subgroup analyses. Such studies could formally test whether Black tax mediates income’s relationship with well-being specifically for Black employees, whether caregiving burdens disproportionately affect Black and Mixed ancestry women, and whether masculine norm constraints operate differently across racial groups.

Second, longitudinal research is needed to examine how well-being changes over time and how interventions moderate trajectories. For example, does introducing flexible scheduling reduce the gender gap in mental well-being? Do financial wellness programmes that acknowledge Black tax reduce financial strain for Black professionals?

Third, development and validation of culturally specific measures that assess constructs such as Black tax financial strain, code-switching demands, and masculine norm conformity stress would enhance construct validity. Existing well-being scales were developed in Western contexts and may not adequately capture culturally specific stressors.

Fourth, multi-level research examining between-organisation variation in well-being disparities could identify which organisational practices promote equity. Do organisations with robust caregiver support policies show smaller gender gaps in mental well-being? Do organisations with culturally sensitive financial wellness programmes show different income–wellbeing relationships for Black versus White employees?

Fifth, intervention research testing whether targeted supports reduce intersectional burdens and improve well-being equity is needed. Randomised controlled trials could test whether caregiver-focused interventions, financial wellness programmes acknowledging Black tax, or masculine norm-challenging peer support groups improve well-being outcomes for specific employee subgroups.

## Conclusion

Workplace well-being in South Africa is shaped by intersecting social determinants rather than isolated demographic categories. Quantitatively, women reported lower mental well-being, and income emerged as the most powerful structural determinant, predicting mental well-being, life satisfaction, and perceived stress. However, qualitative findings revealed the mechanisms underlying these patterns: gendered unpaid labour and caregiving strain constrained women’s mental well-being, kin-based financial obligations (Black tax) appeared to constrain income’s protective effects for Black professionals, and restrictive masculine norms masked men’s hidden distress.

The findings reinforce the need for culturally responsive and equity-oriented workplace mental health strategies that address structural burdens, redistribute resources, and create psychologically safe environments for all employees. Universal well-being interventions that ignore intersectional positioning risk reinforcing disparities by providing resources that are more accessible to already-advantaged groups.

The study demonstrates that workplace mental health equity is achievable when organisations attend to the lived realities of employees positioned differently within social structures, recognising that well-being is not simply an individual responsibility but a product of upstream conditions, intersectional positioning, and organisational systems.

The finding that income predicts well-being but is constrained by Black tax carries a direct theoretical implication: financial resources cannot be treated as uniformly protective in structurally unequal settings. When cultural obligations systematically redirect income through kin networks, salary parity does not produce psychological benefit parity. This challenges resource-based frameworks to move beyond individual-level resource accounting and attend to the social conditions that determine whether resources reach and remain with the individual.

## Data Availability

The data analysed in this study is subject to the following licenses/restrictions: de-identified quantitative survey data (*n* = 87) are available from the corresponding author upon reasonable request, subject to ethical approval and data sharing agreement. Qualitative interview data (field notes, transcripts) cannot be made publicly available due to: (1) participant confidentiality commitments in a small organisational setting where individuals could be identifiable despite anonymization; (2) ethical approval restrictions protecting employee privacy given the insider-researcher context and sensitive topics discussed (workplace criticism, mental health disclosure, racial inequities); and (3) organisational privacy protections for internal workplace data. The manuscript includes sufficient detail (demographic tables, anonymised quotes, thematic summaries) to substantiate all claims and enable replication of the analytical approach. Requests to access these datasets should be directed to anuragshekhar.email@gmail.com.
